# Sex-specific-differences in cardiometabolic risk in type 1 diabetes: a cross-sectional study

**DOI:** 10.1186/1475-2840-12-78

**Published:** 2013-05-24

**Authors:** Alexandra Kautzky-Willer, Kathrin Stich, Juliane Hintersteiner, Alexander Kautzky, Majid Reza Kamyar, Johannes Saukel, Julienne Johnson, Rosa Lemmens-Gruber

**Affiliations:** 1 Gender Medicine Unit, Department of Internal Medicine III, Division of Endocrinology and Metabolism, Medical University of Vienna, Waehringer Guertel 18-20, 1090, Vienna, Austria; 2 Department of Pharmacology and Toxicology, University of Vienna, Vienna, Austria; 3 Department of Pharmacognosy, University of Vienna, Vienna, Austria; 4 Strathclyde Institute of Pharmacy and Biomedical Sciences, Strathclyde University, Glasgow, UK

**Keywords:** Type 1 diabetes mellitus, Gender, Diabetic complications, Cardiovascular risk, Blood pressure, Lipid profile

## Abstract

**Background:**

Little is known about the impact of sex-specific differences in the management of type 1 diabetes (T1DM).

Thus, we evaluated the influence of gender on risk factors, complications, clinical care and adherence in patients with T1DM.

**Methods:**

In a cross-sectional study, sex-specific disparities in glycaemic control, cardiovascular risk factors, diabetic complications, concomitant medication use and adherence to treatment recommendations were evaluated in 225 consecutive patients (45.3% women) who were comparable with respect to age, diabetes duration, and body mass index.

**Results:**

Although women with T1DM had a higher total cholesterol than men, triglycerides were higher in obese men and males with HbA1c>7% than in their female counterparts. No sex differences were observed in glycaemic control and in micro- or macrovascular complications. However, the subgroup analysis showed that nephropathy was more common in obese men, hyperlipidaemic women and all hypertensive patients, whereas peripheral neuropathy was more common in hyperlipidaemic women. Retinopathy was found more frequently in women with HbA1c>7%, obese men and in both sexes with a long duration of diabetes. The multivariate analysis revealed that microvascular complications were associated with the duration of disease and BMI in both sexes and with hyperlipidaemia in males. The overall adherence to interventions according to the guidelines was higher in men than in women. This adherence was concerned particularly with co-medication in patients diagnosed with hypertension, aspirin prescription in elderly patients and the achievement of target lipid levels following the prescription of statins.

**Conclusions:**

Our data showed sex differences in lipids and overweight in patients with T1DM. Although glycaemic control and the frequency of diabetic complications were comparable between the sexes, the overall adherence to guidelines, particularly with respect to the prescription of statins and aspirin, was lower in women than in men.

## Background

Type 1 diabetes (T1DM) is a chronic disease that is still a challenge for both patients and their physicians. Patient education and self-empowerment, good metabolic control and cardiovascular risk management are important to protect from the early development of diabetic complications and increased mortality. Recently, a doubling of new cases of T1DM in European children younger than 5 years and a rise by 70% in the prevalent cases younger than 15 years between 2005 and 2020 was predicted [1]. Therefore, better knowledge about risk factors and the needs of these patients as well as adequate health-care resources to meet these needs are necessary.

Contrary to type 2 diabetes, which is usually characterised by overweight/obesity and increasing age, the literature on the potential sex and gender differences in type 1 diabetes concerning cardiovascular risk factors, metabolic control and drug therapy is scarce [2-4]. Data from the EURODIAB PCS recently showed that women with type 1 diabetes had higher total cholesterol concentrations and that higher triglyceride but lower HDL cholesterol levels were predictors of coronary heart disease in women [5]. In contrast, sialic acid and fibrinogen were strong predictors of CHD only in men with type 1 diabetes beyond the effect of the established risk factors [6]. Data from a German database [7] revealed that female gender, age, duration of diabetes and minority status were significantly associated with poor glycaemic control. However, most studies in T1DM have investigated children or patients at a young age.

Therefore, we aimed to investigate in a cross-sectional study whether gender differences are relevant between male and female adult patients with T1DM with regard to metabolic control, cardiovascular risk factors, the presence of the metabolic syndrome, micro- and macrovascular diabetic complications, drug therapy and adherence to clinical recommendations.

## Methods

### Study design and patients

This study was a cross sectional survey of patient data from medical records and patient questionnaires and interviews to determine the adherence to a medication assessment tool. This study was approved by the Ethics Committee of the Medical University of Vienna and conducted in accordance with the Helsinki Declaration. In total, 225 patients (45.3% women and 54.7% men) with T1DM, attending the diabetes outpatient clinic at the Medical University of Vienna between March 2009 and August 2009, fulfilled the inclusion criteria (age ≤75 years, T1DM, a documented history of the presence or absence of coronary heart disease, obese and non-obese diabetics aged ≥18 years, and having given informed consent). The required sample size was calculated using standard formulae for sampling for a survey to produce percentage frequency rates of nominal data within conventionally acceptable error rates (margin of error 5%) and 95% CIs. Sampling was performed using standard data collection. A previously described questionnaire was used to obtain information about age, known duration of T1DM, height, weight, adherence to drug treatment, smoking habits, alcohol consumption, parental history of diabetes, blood pressure, glycaemic control, lipid profile, and parameters of liver and kidney function [8,9]. In addition, the presence of diabetic microvascular and macrovascular complications and a history of previous percutaneous transluminal coronary angioplasty (PTCA) or coronary artery bypass surgery (CABG) were assessed. The information about life-style parameters, familial predisposition for cardiovascular disease (CVD) and parental history of diabetes was self-reported, but all data concerning weight, height, duration of diabetes, medical history and clinical characteristics were immediately confirmed and completed using the clinical records.

All patients maintained a stable weight, and moderate physical activity and nutrition therapy were recommended for all.

### Subgroup analyses

**Figure 1:**
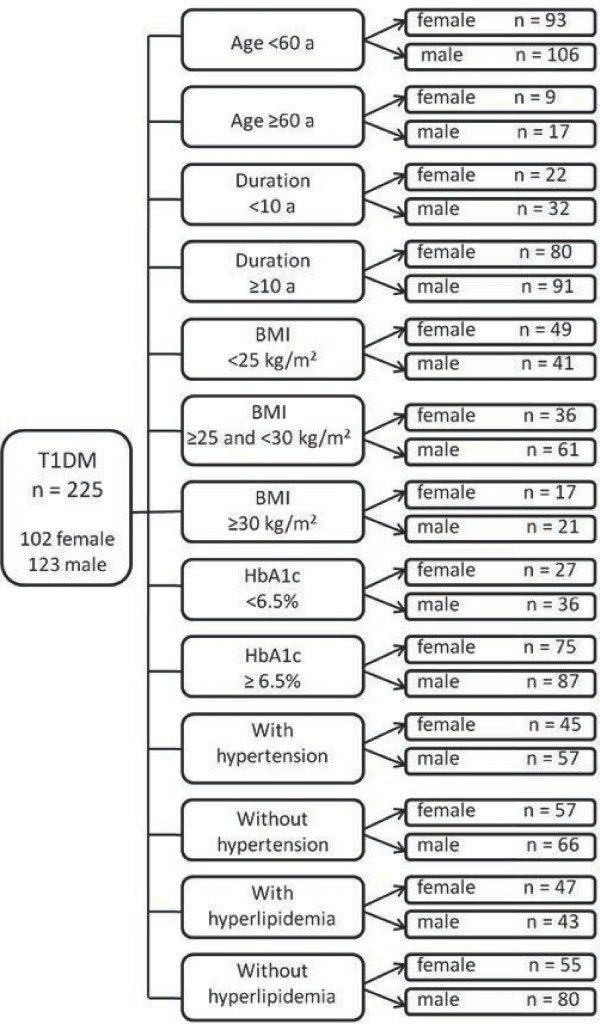
Description of the subgroups.

To identify criteria for which adherence was statistically significant among the subgroups, we classified patients according to age, duration of disease, body weight, glycaemic control, co-morbidities and gender (Figure 1). The subgroup analyses were performed to compare the above-mentioned criteria. Patients with CVD, defined as ischaemic heart disease, myocardial infarction, and/or angina pectoris, stroke and transient ischaemic attacks were considered to require secondary prevention.

Metabolic syndrome (MetS) was defined according to the European Guidelines on cardiovascular disease prevention [10] and the World Health Organization criteria [11] by the presence of diabetes mellitus (DM)/insulin resistance plus ≥2 of the following parameters: obesity (body mass index [BMI] ≥30 kg/m^2^), hypertension (blood pressure [BP] ≥140/90 mmHg or the use of antihypertensive drugs), and dyslipidaemia (triglycerides [TG] ≥1.71 mmol/L and/or HDL-C <0.9 mmol/L for men and <1.03 mmol/L for women). Insufficient glycaemic control was defined as HbA1c >7%, and poor glycaemic control as HbA1c >8%.

### Adherence to guidelines and medication assessment tool

To test the adherence of the patient populations to evidence-based clinical prescribing recommendations, a medication assessment tool (MAT) was employed [8,12]. This instrument is based on the guidelines established by the Scottish Intercollegiate Guidelines Network [13], which is in accordance with the guidelines of the American Diabetes Association and the European Society of Cardiology [14,15]. Furthermore, a review of NICE guidelines 43 [16], 34 [17], and 48 [18] and of SIGN guideline 97 [19] was performed. The final medication assessment tool comprised 30 criteria including 13 criteria for general cardio-preventive measures, 10 for hypertension, 3 for diabetes management, and 4 for anti-obesity medication. For each patient, each criterion item was judged as “applicable”, “insufficient data” (lack of information), “not applicable” (criterion relevant for patient but the patient’s data did not meet the qualifying statement) or “justified non-adherence” (explanation for the patient’s treatment not meeting a quality criterion). Adherence to the guideline recommendations was calculated as previously described in detail [8,12]. Adherence above 70% was arbitrarily judged as a high level of adherence, between 50 and 69% as intermediate and below 50% as low.

### Statistics

The levels of adherence were compared using the χ^2^ test and *P*<0.05 as the threshold for statistical significance. A Microsoft Access (Microsoft Corporation, Redmond, WA) database was created, from which the data from the specific subgroups were extracted. These data were statistically evaluated using SPSS 16.0 (SPSS Inc., Chicago, IL).

For metric and ordinal characteristics, the number of patients and arithmetic means with standard errors are given. For the evaluation of statistically significant sex-dependent differences within the whole sample and specific subgroups, Student’s *t* test, Welch t test and Mann–Whitney U test were used, depending on the sample size and test criteria. For nominal characteristics, the number of patients and percentages are given. Statistically significant sex-dependent differences for the whole sample and specific subgroups were calculated using the χ^2^ test and the Fisher exact test. Statistical significance was determined at levels of *P*<0.05, *P*<0.01, and *P*<0.001.

The multivariate discriminant function analysis tool (©Statistica 10.0, StatSoft, Germany) was used to determine which variables discriminate between healthy and sick persons for the three important complications: nephropathy, peripheral neuropathy, and retinopathy. We used the so-called forward stepwise analysis. In this type of analysis, a model of discrimination is built step-by-step. Specifically, at each step, all variables are reviewed and evaluated to determine which one will contribute most to the discrimination between groups. The stepwise procedure is “guided” by the respective *F* to enter and *F* to remove values. The *F* value for a variable indicates its statistical significance in the discrimination between groups, that is, it is a measure of the extent to which a variable makes a unique contribution to the prediction of group membership. The generated classification matrices provide evidence of correctly assigned patients.

The general regression model tool (©Statistica 10.0) was used to analyse the multivariate regression of 11 continuous variables (duration of T1DM, systolic and diastolic blood pressure, blood glucose, HbA1c, BMI, total cholesterol, LDL, HDL, total cholesterol/HDL ratio, and triglycerides) against the ordinal arranged combination of the three binary variables - nephropathy (1), peripheral neuropathy (2), and retinopathy (3) according to the formula (1 + 2) + (1 + 3) + (2 + 3). The model was built up with the tool combination multiple regression (method best subsets) + ordinal multinomial distribution + the logit link function. One major output is an ordered list of the so-called Wald statistic of the used variables with the amount of the power and probability of the prediction of the ordinal multinomial variable.

## Results

### Metabolic syndrome and patient profile

MetS affected 13.5% of the entire study sample (Table 1). Sex-dependent differences were observed regarding hyperlipidaemia, with more women having hyperlipidaemia with a significantly higher total cholesterol and HDL-cholesterol concentration compared with men (Table 1). This finding of significantly higher total cholesterol levels in women was observed in all subgroups (see Figure 1) except in the overweight patients and patients ≥60 years. Especially in the subgroups of obese and hypertonic women, not only was total cholesterol significantly higher than in their respective male subgroups (220.8 [54.1] vs. 183.7 [41.2] mg/dl, *P*<0.05, and 206.2 [42.6] vs. 185.9 [34.6] mg/dl, *P*<0.05, respectively) but LDL cholesterol was also significantly higher (135.0 [48.5] vs. 98.0 [34.8], *P*<0.05, and 114.5 [39.2] vs. 100.1 [27.8] mg/dl, *P*<0.05). Remarkably, compared with the whole female sample and other female subgroups, HDL cholesterol in obese women was lowest at 62.8 [23.0] mg/dl. Consequently, this lower HDL cholesterol value resulted in an increase in the total cholesterol/HDL ratio, which was higher in men than in women in the total sample (Table 1) and in all other subgroups. In obese patients, however, the total cholesterol/HDL ratio of 3.65 [1.15] for women was not different from that of men (3.64 [0.91]). Furthermore, in all male subgroups, triglycerides were elevated and higher than those in their female counterparts, particularly in obese men (172.3 [75.2] vs. 115.08 [71.2], *P*<0.05) and in male patients with insufficient glycaemic control (132.9 [68.7] vs. 109.7 [63.3], *P*<0.05).

**Table 1:** Demographic data and clinical characteristics of T1DM patients

**Characteristics**	**Women**	**Men**
	**(n=102)**	**(n=123)**
Age (years)	41.3±13.6	43.1±13.9
Percentage of geriatric patients (>60 years)	8.8	13.8
Age (years) at diagnosis	19.2±10.2	21.4±11.5
Duration (years) of disease	19.3±7.7	17.3±10.4
Smokers (%)	29.1	36.3
Alcohol abstinence (%)	53.5^§§§^	20.4
Metabolic syndrome (%)	12.6	14.3
Blood glucose (mg/dl)	122.9±50.4	123.8±56.4
HbA1c (%)	7.6±1.0	7.5±1.1
HbA1c<6.5% (%)	26.5	29.3
Hypertension (%)	44.2	46.7
Systolic blood pressure (mmHg)	135.5±18.9	136.9±17.4
Diastolic blood pressure (mmHg)	82.7±12.3	82.9±8.7
BMI (kg/m^2^)	25.5±4.9	26.3±3.4
Overweight (%)	33.3	44.7^§§§^
Obese (%)	12.7	13.0
Hyperlipidaemia (%)	46.3	35.2
Total cholesterol (mg/dl)	203.1±38.3	186.7±33.6**
HDL cholesterol (mg/dl)	70.6±19.9	59.1±15.9***
LDL cholesterol (mg/dl)	111.1±32.2	103.4±28.2
Total cholesterol/HDL ratio	3.3±1.6	3.6±1.4
Triglycerides (mg/dl)	106.6±69.1	124.6±71.8

Data are shown as mean±SE.

^§§§^ P < 0.001 by χ^2^ test, ** P < 0.01 by t test, *** P < 0.001 by t test.

### Diabetic complications

**Figure 2:**
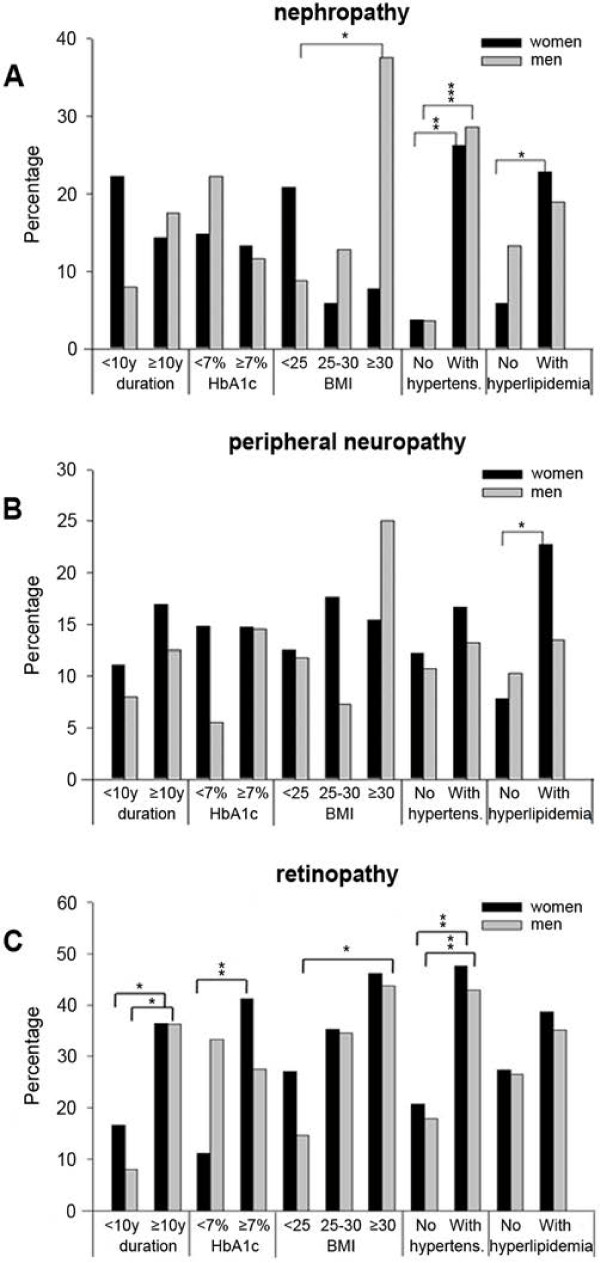
**Percentage of diabetic complications in the presence and absence of particular risk factors.** The percentage of women and men with nephropathy (**A**), peripheral neuropathy (**B**) and retinopathy (**C**) is shown for several patient subgroups. The significance of the differences between patient subgroups in the presence and absence of particular risk factors was calculated and is indicated by asterisks (* *P*<0.05, ** *P*<0.01, *** *P*<0.001). **2A:** The risk for the development of nephropathy increased significantly in obese men, hyperlipidaemic women and hypertensive male and female patients compared with normal weight men, normolipidaemic women and normotensive male and female patients. **2B:** There was a significantly more frequent occurrence of peripheral neuropathy in hyperlipidaemic women than in normolipidaemic women. **2C:** Retinopathy was observed significantly more often in male and female patients with a longer duration of disease, in women with poor glycaemic control, in obese men, and in hypertensive men and women compared with patients in the respective subgroups without these risk factors.

In the entire sample, no significant sex-dependent differences for the incidence of micro- and macrovascular complications were observed (Table 2). Macrovascular diseases occurred rarely in our sample of T1DM patients, whereas microvascular complications were observed more frequently (Table 2). They already occurred at an earlier stage of T1DM. Nephropathy, peripheral neuropathy and retinopathy were found in all T1DM patients in both the presence and absence of other risk factors. The univariate analysis highlights an increased risk for the development of nephropathy in obese men, hyperlipidaemic women and hypertensive men and women (Figure 2A, Table 3). A significantly increased risk for peripheral neuropathy was only observed in hyperlipidaemic women (Figure 2B, Table 3). The threat for retinopathy was significantly higher in men and women suffering from T1DM more than 10 years, in women with insufficient glycaemic control, and in hypertensive patients (Figure 2C, Table 3). Obese men had a significantly higher risk to develop retinopathy than normal weight males. However, there were no significant differences between obese and overweight patients or between normal weight and overweight patients.

**Table 2:** Percentage of men (n=123) and women (n=102) with diabetic complications

**Micro- and macrovascular diseases**	**Women**	**Men**	**P-value**
Familial predisposition for CVD (%)	25.3	16.2	0.112
Nephropathy (%)	13.7	15.2	0.755
Peripheral neuropathy (%)	14.7	11.4	0.487
Retinopathy (%)	32.6	29.5	0.635
Peripheral artery occlusive disease (PAOD) (%)	3.2	2.9	1.000
PTCA/CABG (%)	4.2	1.0	0.193
Angina pectoris (%)	0	1.9	0.499
Cerebral ischemia (%)	2.1	1.0	0.475
Myocardial infarction (%)	1.1	0	0.605

Cardiovascular disease (CVD), Percutaneous Coronary Transluminal Angioplasty/Coronary artery bypass grafting (PCTA/CABG).

**Table 3:** Evaluation of risk for retinopathy, nephropathy and/or peripheral neuropathy by multivariate analyses

**Parameter**	**Univariate analysis**						**Multivariate discriminant function analysis**						**Multivariate regression analysis**	
	**(P-values)**						**(P-values)**						**(P-values)**	
	***Retinopathy***		***Nephropathy***		***Neuropathy***		***Retinopathy***		***Nephropathy***		***Neuropathy***		***Microvascular diseases***	
	**m**	**f**	**m**	**f**	**m**	**f**	**m**	**f**	**m**	**f**	**m**	**f**	**m**	**f**
**Duration T1DM**	**0.021**	**0.043**	*0.253*	*0.553*	*0.079*	*0.098*	**0.000**	**0.000**	*0.330*	*0.534*	**0.049**	**0.041**	**0.000**	**0.001**
**BMI**	**0.036**	*0.312*	**0.028**	*0.299*	*0.249*	*1.000*	**0.025**	*0.635*	*0.109*	**0.005**	*0.298*	*0.467*	**0.011**	**0.039**
**Total cholesterol**	*0.352*	*0.246*	*0.439*	**0.017**	*0.750*	**0.041**	**0.021**	**0.019**	**0.017**	*0.426*	*0.958*	*0.522*	**0.002**	*0.666*
**LDL cholesterol**	*0.252*	**0.031**	*0.393*	**0.052**	*0.264*	*0.500*	*0.184*	**0.022**	*0.074*	**0.015**	*0.465*	*0.537*	**0.023**	*0.084*
**HDL cholesterol**	*0.405*	**0.032**	**0.052**	*0.383*	*0.247*	*0.659*	*0.106*	**0.018**	**0.038**	*0.589*	*0.695*	*0.511*	**0.002**	*0.640*
**Triglycerides**	**0.049**	**0.049**	*0.421*	**0.040**	*0.149*	*0.169*	**0.009**	**0.029**	*0.178*	*0.273*	*0.263*	*0.578*	**0.007**	*0.732*
**Syst./Diast. RR**	**0.005**	**0.006**	**0.000**	**0.002**	*0.806*	*0.637*	*0.940*	*0.141*	*0.223*	*0.797*	*0.657*	*0.867*	*0.621*	*0.483*
**HbA1c**	*0.536*	**0.005**	*0.150*	*0.998*	*0.212*	*1.000*	*0.315*	*0.082*	*0.780*	*0.265*	*0.238*	*0.315*	*0.381*	*0.453*

Men (m), Women (f), Systolic/Diastolic blood pressure (Syst./Diast. RR).

Hence, a multivariate discriminant function analysis was conducted to determine which parameters discriminate between diabetic patients without microvascular diseases and those suffering from neuropathy, peripheral neuropathy or retinopathy. Our data confirmed the pivotal role of the duration of diabetes for the onset and progression of retinopathy and neuropathy (Table 3). In addition, in our sample, diabetic retinopathy was significantly associated with dyslipidaemia (males and females) and body weight (males) (Table 3). The multivariate regression analysis of eleven parameters against the three variables of nephropathy, peripheral neuropathy and retinopathy revealed that the duration of disease and body weight in male and female patients and dyslipidaemia in males were joint risk factors for the development of microvascular diseases.

### Pharmacotherapy

The adherence to interventions according to the respective guidelines is listed in Tables 4, 5 and 6, ranked from highest to lowest adherence. Interestingly, the overall adherence was higher in men (56.9 [47.1-62.7]) than in women (46.5 [37.1-55.9]) (*P* = 0.0573).

**Table 4:** Gender-dependent adherence to general cardio-preventive criteria

**Criteria**	**Adherence (%) [95% Cl]**	
	**Men**	**Women**
	**n=123**	**n=102**
**High level of adherence to general cardio-preventive measures**		
• Patient with apparent contraindication/intolerance to aspirin therapy is prescribed clopidogrel 75 mg	100	100
• Prescribing of angiotensin-converting enzyme inhibitors (ACE-Is) or angiotensin II receptor blockers (ARBs) post myocardial infarction	100	100
• Prescribing of beta-blocker in post myocardial infarction patients or in patients with coronary heart disease	68.8 [56.7-80.9]	88.0* [76.1-97.9]
• Prescribing of ACE-I/ARB in patients with microalbuminuria/proteinuria	76.1 [63.8-88.4]	75.7 [61.9-89.5]
**Intermediate level of adherence to general cardio-preventive measures**		
• Patient aged ≥40 years is prescribed a statin when pretreatment serum cholesterol was ≥200 mg/dl	67.4 [57.7-77.2]	60.9 [50.7-71.2]
**Low level of adherence to general cardio-preventive measures**		
• Patient who is described a statin has achieved a triglyceride level of 350 mg/dl and LDL level of 80 mg/dl	40.3 [28.1-52.5]	19.6** [4.0-24.8]
• Patient aged ≥50 years is prescribed aspirin	53.4 [42.0-64.9]	37.1* [25.8-48.5]
• Patients aged <50 years with cardiovascular risk factors is prescribed aspirin	5.9 [0–17.1]	0
• Patient who continues to smoke has been offered smoking cessation advice which involves structured behavioural support plus nicotine replacement therapy or bupropion/varenicline	0	0

* P < 0.05, ** P < 0.01.

**Table 5:** Gender-dependent adherence to hypertension criteria

**Criteria**	**Adherence (%) [95% Cl]**	
	**Men**	**Women**
	**n=123**	**n=102**
**High level of adherence to hypertension criteria**		
• Prescribing of antihypertensive drug(s) in hypertensive diabetic patient	100	100
• No co-prescribing of thiazide + beta blocker in treated hypertension	100	100
• Patient diagnosed with hypertension has a treatment plan that does NOT include oral contraceptives, corticosteroids, NSAIDs, high sodium containing products, sympathomimetics, monoamine oxidase inhibitors	96.9 [92.6-100]	84.6* [74.8-94.5]
• Addition of a calcium channel blocker (CCB) and/or diuretic in patients whose blood pressure remains uncontrolled with ACE-Is or ARBs	89.7 [80.2-99.3]	87.8 [78.6-96.9]
• Prescribing of an ACE-I or ARB in hypertensive patient	85.9 [74–94.5]	87.1 [78.8-95.4]
**Low level of adherence to hypertension criteria**		
• Achievement of blood pressure target in patients on aspirin	34.1 [20.1-48.1]	23.1 [6.9-39.3]
• Patient with treated hypertension and with co-existing kidney, eye or cerebrovascular damage and/or with two or more features of MetS has achieved blood pressure control of ≤130/80 mmHg	16.4 [7.1-25.7]	10.2 [2.5-17.9]

* P < 0.05.

**Table 6:** Gender-dependent adherence to diabetes management

**Criteria**	**Adherence (%) [95% Cl]**	
	**Men**	**Women**
	**n=123**	**n=102**
**High level of adherence to diabetes management**		
• Prescribing of insulin	100	100
• Test blood glucose themselves	100	100
• Patients with a diagnosis of DM of at least 15 months has had two HbA1c measurements taken at least twice within the past 15 months	92.9 [87.9-98]	91.4 [85.7-97.1]
**Intermediate/Low level of adherence to diabetes management**		
• Achievement of HbA1c<6.5% in patients on insulin	56.7 [45.2-68.2]	43.4* [32.8-54.0]

* P < 0.05.

Low levels of adherence were found with respect to advice for smoking cessation in patients who continued smoking (Table 4), in the prescription of aspirin (Tables 4 and 5), and in the achievement of target LDL cholesterol, triglyceride (Table 4), and blood pressure levels (Table 5) and HbA1c<6.5% (Table 6). Both genders indicated low adherence to the criterion “Patient who is prescribed a statin has achieved target cholesterol and triglyceride levels”, but females showed even significantly (*P* < 0.01) lower levels than males (Table 4). Furthermore, female patients older than 50 years were prescribed aspirin significantly less frequently (*P <* 0.05) than their male counterparts. Similarly, in male patients aged <50 years with prominent risk factors, such as a family history of CVD, smoking and MetS, only a very low adherence (5.9% [0–17.1]) for the prescription of aspirin was estimated. However, for female patients, the adherence to this criterion was zero in our sample. In addition, the achievement of a target HbA1c<6.5% was significantly different between men (intermediate adherence) and women (with low adherence) (*P*<0.05) (Table 6), whereas no significant sex-dependent difference in the patients with insufficient glycaemic control was observed.

High levels of adherence were observed for all other cardio-preventive and hypertension criteria and for diabetes management. Although both genders indicated high adherence (92.9% [79.4-100]) to the criterion “Patient diagnosed with hypertension has a treatment plan that does not include oral contraceptives, corticosteroids, non-steroidal anti-inflammatory drugs (NSAIDs), high sodium containing products, sympathomimetics and/or monoamine oxidase inhibitors”, the female population showed significantly (*P*<0.05) lower levels (84.6%) than their male counterparts (96.9%) (Table 5). Among the women who had no appropriate treatment plan according to the above mentioned criterion, 4.8% had been prescribed postmenopausal hormone replacement therapy, which was equalised to oral contraceptives. In addition, 12.8% of women had been prescribed a corticosteroid, and 1.5% an NSAID. In most cases, a combination of antihypertensive drugs was administered, including diuretics, ACE inhibitors, angiotensin II type 1 receptor blockers, calcium antagonists, α1-blockers, and α2-agonists. In contrast, the prescription of beta-blockers in post-myocardial infarction patients or patients with coronary heart disease was significantly lower in men than in women (68.8% [56.7-80.9] vs. 88.0% [76.1-97.9], P<0.05). For all other applied criteria concerning cardio-preventive measurements and the management of hypertension and T1DM, no significant differences between men and women were observed (Tables 4, 5 and 6).

## Discussion

The T1DM incidence is comparable between men and women in most populations [20,21] and is linked to long-term mortality [22,23], particularly in females with T1DM diagnosed before the age of 30 years [24]. Childhood-onset T1DM with at least 25 years of follow-up showed no sex-differences in the survival and mortality trends [25]; however, these female patients were 13 times more likely to die than age-matched women in the general population, whereas these male patients only had a 5-fold increased mortality rate compared with the male background population. Similar data stratified by sex were also reported from Norway [22] and the Diabetes UK Cohort Study [26]. Thus, although young women are usually protected from cardiovascular disease and nephropathy compared with males, women with T1DM appear to be a very vulnerable group of patients. The female sex was speculated to completely lose its general survival advantage in diabetic subjects, and increasing effects on the long-term complications and potential differences in treatment may contribute to this finding [25].

Therefore, in this analysis, we studied sex differences regarding metabolic control, complications and pharmacological treatment with regard to the adherence to guidelines in a characteristic sample of T1DM patients treated at the outpatient department of a university clinic. The sample consisted of a homogeneous group of Central European patients. They were middle-aged with a quite long duration of disease and onset after puberty in most cases. Most were normal-weight or moderately overweight, and less than 15% of men and women fulfilled the criteria of metabolic syndrome, which is lower than the rate usually found in subjects of comparable age in Europe [27]. However, the rate was only slightly higher and thus comparable with that found in Italian adolescents with T1DM [28]. Interestingly, in these young patients, women had a higher rate of abdominal adiposity, and male adolescents had higher mean systolic blood pressure values, which were not observed in our cohort. In accordance with our results, HbA1c was only slightly but insignificantly higher in the female patients. In another study, insulin resistance and increased carotid intima-media thickness characterised adolescent T1DM patients without the characteristics of MetS, suggesting that insulin resistance and early atherosclerosis may already be present in lean T1DM patients [29]. Similarly, in a longitudinal study in children and adolescents with T1DM, the carotid intima-media thickness was elevated compared with the healthy controls, and these patients further progressed in subclinical atherosclerosis during a four year period [30]. Blood pressure and BMI were related to its increment, with no gender differences in the carotid intima-media thickness values. In the SEARCH CVD study age, sex, adiposity and systolic blood pressure were determinants of increased IMT in youth with T1DM, but after adjustment of HbA1c these differences disappeared emphasizing the importance of good glycaemic control [31].

Several studies confirmed worse glycaemic control in female diabetic subjects, but most studies referred to elderly patients with T2DM. Data from a German database showed that female gender, age, duration of diabetes and minority status was significantly associated with poor glycaemic control in T1DM [7]. Similarly, a cross-sectional analysis of the Diabetes Control and Complications Trial/Epidemiology of Diabetes Interventions and Complications (DCCT/EDIC) cohort reported that women were less likely than men to achieve HbA1c<7.0%, whereas the achievement of target lipid levels was not significantly different between the sexes [32]. In contrast, the gender-specific characterisation of children and adolescents with T1DM in Austria and Germany showed differences in the distribution of cardiovascular risk factors in disfavour of females, including higher cholesterol levels [33]. In our study, women showed higher mean total cholesterol levels than men, and overall, women tended to feature hyperlipidaemia more often than their male counterparts. In the obese and hypertonic subgroups, LDL cholesterol was also higher in the women than in the men. In contrast, triglycerides were higher in men than in women but only in the obese group, and in those patients with insufficient glycaemic control, this difference became significant. Similar findings were observed in another, smaller longitudinal study in patients with T1DM at our institution [34] and in many studies on T2DM patients [9]. Moreover, data from the EURODIAB PCS recently clearly showed that women with T1DM had higher total-cholesterol concentrations than their male counterparts [5].

The cross-section Brazilian Type 1 Diabetes Study reported complex and heterogeneous relationships between glycaemic control and fasting lipids but low HDL cholesterol consistently related to exogenous hyperinsulinaemia [35]. However, the analyses were not performed separately by gender.

US adults with diabetes were shown to be at lower short-term CVD risk than previously assumed with proportionately fewer high-risk persons among the females and those with T1DM by using global risk assessment equations for total CVD [36]. However, almost 30% had pre-existing CVD without sex differences. It should be mentioned that most risk scores were derived from predominantly male cohorts and that the risk may be underestimated in female diabetic patients. In addition, this study reported that less than 15% of all diabetic patients simultaneously achieved their HbA1c, blood pressure and cholesterol goals. With respect to diabetic complications, no sex differences were found in the rates of micro- or macrovascular complications in general (Figure 1A-C; Table 2); in both sexes, retinopathy was most prevalent followed by nephropathy, whereas cardiovascular diseases tended to be higher in women than in men without reaching statistical significance as only a few events were documented in this small cohort in both sexes. These events occurred only after a disease duration of more than 10 years. However, the absence of a gender difference in CVD with even more events in women clearly supports the notion that diabetes reverses the sex-related risk relationship in the non-diabetic population and that diabetes is particularly harmful in women. Although the reason for these sex-dimorphic effects in diabetes is not entirely clear at present, there are several hypothesis underlining the importance of sex-specific risk factors. In adolescents with T1DM but not in healthy subjects, girls had a higher per-cent of trunk fat compared with boys, whereas other cardiovascular risk factors, including vascular elasticity, did not differ in children with or without diabetes [37]. Thus, more centrally distributed fat could also contribute to the relatively higher cardiovascular risk in females with T1DM. Of note, higher trunk fat mass was associated with a higher prevalence of coronary heart disease only in female T1DM in the Pittsburgh Epidemiology of Diabetes Complications cohort [38]. However, a higher stiffness of the central elastic arteries was also found in older female T1DM patients compared with their male counterparts [39]. The Coronary artery calcification in type 1 diabetes study showed that T1DM increases the prevalence and severity but reduces the gender differences of coronary artery calcification [40]. Gender differences in insulin resistance-associated fat deposition and in HDL and LDL cholesterol distribution may explain why type 1 diabetes increases coronary calcification in women relatively more than in men. Furthermore, female sex hormones, particularly oestrogen, may exert beneficial effects on the cardiovascular system and energy metabolism, which could be impaired in diabetic women potentially via an unfavourable oestrogen receptor balance and distribution [41]. In fact, diabetes status has been shown to abolish the vascular protective effect of oestrogen in female rats [42]. This lack of the protective effects of oestrogen was mainly ascribed to its failure to reverse the impaired basal release of NO and the abnormal relaxation to histamine in the aorta of diabetic female rats. Furthermore, this risk could be related to dietary fat intake and lifestyle [10]. Altogether in humans, the relationship among sex hormones, gender, diabetes and vascular disease is complex and not fully understood. Given that this “diabetes gender paradox” also refers to T1DM, the importance of hyperglycaemia in addition to other cardiovascular risk factors that usually accompany T2DM is further suggested.

Regarding microvascular complications, among the hyperlipidaemic subgroups, women had a much higher rate of neuropathy than those with normal lipids. Nephropathy was highest in obese males, in hyperlipidaemic females and in hypertensive patients of both sexes. Many other studies found the male sex to be a risk factor for nephropathy, which accompanies a potential protective effect of oestrogen that was shown in experimental models of retinopathy and nephropathy [43,44]. An increased risk of retinopathy was observed in both sexes with a disease duration longer than 10 years, obesity and hypertension, whereas insufficient glycaemic control was associated with a higher rate in women but not in men. Diabetic retinopathy is the major diabetic microvascular complication in T1DM [45] and may also increase the risk for all-cause mortality and incidence of CVD [46]. The presence of cardiovascular risk factors explained the associations to a large extent, except for the association with proliferative retinopathy, which suggests that other shared mechanisms may be involved. In the Prospective German Diabetes Documentation System Survey, diabetic retinopathy in T1DM patients [47] was associated with the male sex in addition to established risk factors. However, regarding sex as a risk factor, the results are controversial. Similar to the study by Monti, a recently published cross-sectional population-based study from Finland reported that female sex together with a longer duration of disease, older age and higher HbA1c explained 35% of diabetic retinopathy [48]. Of note, females appeared to have better control but a higher risk of retinopathy. However, there are also reports on a higher risk in male patients [49]. Furthermore, higher testosterone levels were found in patients with proliferative retinopathy [50]. Therefore, the impact of sex on the onset and progression of retinopathy is unclear at present, and in accordance with the data of our small cohort, additional risk factors can be hypothesised to contribute to an increased risk in one or the other sex. Therefore, basic mechanistic studies and longitudinal clinical data in large cohorts are necessary to clarify the impact of sex and gender-related risk factors on the development of diabetic complications.

Intensive diabetes therapy during the DCCT was shown to decrease carotid artery intima-media thickness (IMT) progression and thus atherosclerosis during 12 years of EDIC follow-up compared with conventional therapy [51]. However, only in men was the IMT significantly lower in the intensified therapy compared with the standard therapy after 6 years of follow-up despite similar differences in glycaemic control between both sexes. The beneficial effects of intensified therapy and better HbA1c on IMT during EDIC were partially mediated by the effects on blood pressure and hypertension. In a previous study in patients with T2DM, women had a similar rate of myocardial infarctions and cerebral ischaemia compared with men but a lower rate of coronary interventions and less aspirin therapy [9]. Many studies proved sex differences in the use of antihypertensive, lipid-lowering or antifibrinolytic drug therapies in patients with T2DM [3,52,53], but there are only a few studies in T1DM. In the present study, the overall adherence to pharmacological intervention according to specific guidelines was lower in females than in males in the T1DM subjects. Low adherence was found for the prescription of statins, achievement of treatment goals for lipids, and for the prescription of aspirin in patients with prominent cardiovascular risk younger than 50 years. Again, the adherence was even lower in women than in men. However, no difference was found in the antihypertensive or antihyperglycaemic treatments (insulin regimes) and in the cardioprotective measurements between both sexes. In the DCCT/EDIC cohort, women were also significantly less likely than men to report using aspirin and statins as well as angiotensin-converting enzyme inhibitors or angiotensin II receptor blockers [32], showing that risk-reducing therapies are underused in women with diabetes. Of note in our small cohort of patients with CHD, female patients even had higher prescription rates of beta-blockers than male patients. Therefore, efforts to increase awareness about the cardiovascular risk in diabetic females seems to show some benefits; improving the guideline-based clinical practice and adherence to drug therapy must be a major target in all diabetic patients, independent of sex.

Our study has several limitations. This is a cross-sectional analysis, and therefore no data on the progression of complications or survival are available. The adherence to prescription according to guidelines is objectively documented, but the adherence/compliance of the patient to his/her medications is self-reported and might be overestimated. The sample is relatively small, and thus additional studies in larger cohorts including longitudinal data are recommended to further study the important sex differences in T1DM.

## Conclusions

In summary, our results support the hypothesis that the female sex may lose its general cardiovascular advantage in diabetic subjects as women had comparable rates of micro- and macrovascular complications with even more cardiovascular events. Obesity and dyslipidaemia may exert sex-dimorphic effects on the development of long-term complications, whereas hypertension and a long duration of disease are related to complications in both sexes. Women were less likely to be treated with statins and aspirin and had a worse metabolic situation with higher total cholesterol levels than males with T1DM. Adherence to pharmacological intervention according to the guidelines was lower in women than in men. Therefore, differences in the treatment may contribute to this gender-difference in cardiometabolic risk. A continuing need for improvements in the treatment and care is essential in all patients with T1DM but is particularly striking for female type 1 diabetics.

## Abbreviations

ACE-I: Angiotensin-converting inhibitors; ARB: Angiotensin II receptor blocker; BMI: Body mass index; CABG: Coronary artery bypass surgery; CVD: Cardiovascular disease; DCCT: Diabetes control and complications trial; DM: Diabetes mellitus; EDIC: Epidemiology of diabetes interventions and complications; IMT: Intima-media thickness; MetS: Metabolic syndrome; NSAID: Non-steroidal anti-inflammatory drug; PTCA: Percutaneous transluminal coronary angioplasty; T1DM: Type 1 diabetes; T2DM: Type 2 diabetes.

## Competing interest

The authors declare that they have no competing interests.

## Authors’ contributions

All persons listed as authors contributed substantially to the paper: AKW and RL designed the study and were responsible for reporting of the work. KS, JH and MR were responsible for performance of the study and data analysis. AK contributed to data analysis and writing the paper. JJ contributed to discussion. All authors read and approved the final manuscript.
